# Facts and myths about use of esketamine for treatment-resistant depression: a narrative clinical review

**DOI:** 10.3389/fpsyt.2024.1394787

**Published:** 2024-05-15

**Authors:** Matteo Di Vincenzo, Vassilis Martiadis, Bianca Della Rocca, Eleonora Arsenio, Andrea D’Arpa, Antonio Volpicelli, Mario Luciano, Gaia Sampogna, Andrea Fiorillo

**Affiliations:** ^1^ Department of Psychiatry, University of Campania “L. Vanvitelli”, Naples, Italy; ^2^ Department of Mental Health, Community Mental Health Center DS 25, Azienda Sanitaria Locale Napoli 1 Centro, Naples, Italy

**Keywords:** treatment-resistant depression, esketamine, major depressive disorder, recovery, remission

## Abstract

**Introduction and aims:**

Treatment-resistant depression (TRD) occurs when at least two different antidepressants, taken at the right dosage, for adequate period of time and with continuity, fail to give positive clinical effects. Esketamine, the S-enantiomer of ketamine, was recently approved for TRD treatment from U.S. Food and Drug Administration and European Medicine Agency. Despite proved clinical efficacy, many misconceptions by clinicians and patients accompany this medication. We aimed to review the most common “false myths” regarding TRD and esketemine, counterarguing with evidence-based facts.

**Methods:**

The keywords “esketamine”, “treatment resistance depression”, “depression”, “myth”, “mythology”, “pharmacological treatment”, and “misunderstanding” were entered in the main databases and combined through Boolean operators.

**Results:**

Misconceptions regarding the TRD prevalence, clinical features and predictors have been found. With respect of esketamine, criteria to start treatment, dissociative symptoms, potential addiction and aspects of administration and monitoring, were found to be affected by false beliefs by clinicians and patients.

**Discussion and conclusion:**

TRD represents a challenging condition, requiring precise diagnosis in order to achieve patient’s full recovery. Esketamine has been proved as an effective medication to treat TRD, although it requires precautions. Evidence can inform clinical practice, in order to offer this innovative treatment to all patients with TRD.

## Background

Major Depressive Disorder (MDD) is a severe mental disorder affecting approximately 280 million people worldwide and representing globally the leading cause of disability. MDD has been conceptualized as a syndrome characterized by depressed mood, loss of pleasure and interest, and other affective, cognitive and somatic symptoms persisting for more than two weeks (1–3). Moreover, MDD impairs psychosocial functioning and quality of life (4, 5). A clinical characterization of the individual patient is necessary in order to develop personalized treatment plan with the final aim of reaching the full recovery (6–9). People with MDD report many physical comorbidities, with a negative impact on the long-term quality of life and reducing their life expectancy (10).

Patients suffering from MDD can report a recurrent course of the disorder, with up to 50% of them not experiencing a full recovery after the first episode, and up to 35% experience more than one episode (11). Therefore, based on the longitudinal course of the disorder, several authors have proposed to distinguish difficult to treat depression from treatment-resistant depression (TRD). In particular, it is a clinical condition characterized by lack of response to appropriate treatment. The construct of TRD is very complex, as witnessed by the fact that several definitions have been proposed (12). A consensus definition is still not available, with implications on epidemiology, policy decision-making and clinical utility (13, 14). No single biomarker has been identified so far which can be considered as a benchmark for depression (15, 16) and for TRD, reflecting a common difficulty in findings biomarkers for mental disorders (17–19).

The European Medicine Agency (EMA) defined TRD as a “failure to produce significant clinical results with a treatment of at least two different antidepressants (of the same or different classes) administered at the right doses and for an adequate amount of time, with verified patients’ compliance to treatment” (20). Although this definition focuses only on pharmacological aspects and does not consider psychotherapy as a strategy for mild conditions, it is widely applied in the context of research (21, 22).

Consistently to this conceptualization, EMA approved intranasal esketamine in combination with an SSRI or a SNRI for the treatment of adults with TRD in December 2019 (23), following the lead of U.S. Food and Drug Administration (24). The approval of esketamine for treating TRD has introduced an antidepressant drug with an innovative mechanism of action into clinicians’ armamentarium. According to recent guidelines for managing TRD, several strategies have been suggested, including the combination or switch of antidepressants; augmentation with antipsychotic and/or mood stabilizers (25); administration of intravenous/intranasal ketamine (26) and neurostimulation techniques (electroconvulsive therapy, deep brain stimulation, vagal nerve stimulation, repetitive transcranial stimulation) (27–29).

Esketamine is the S-enantiomer of ketamine, working as non-selective, non-competitive antagonist of N-methyl-D-aspartate (NDMA) receptor (30). Subsequent downstream of glutamate release stimulates the activation of AMPA receptors, by initiating intracellular signaling cascades, resulting in the activation of mammalian target of rapamycin (mTOR) and increase of brain-derived neurotrophic factor (BDNF) levels, with positive effects on synaptic plasticity (31, 32). In terms of pharmacokinetics, intranasal esketamine has mean bioavailability of about 48%, its peak is reached until to 40 minutes from last spray, presents biphasic half-life and undergoes metabolism through CYP-2B6, -3A4, -2C9, -2C19, hydroxylation and glucuronidation (33).

Esketamine may be associated with craving behavior and additional potential (34). Indeed, dissociative state is characterized by depersonalization and derealization (24), while hallucinations have been reported as a consequence of the recreational use of ketamine, not for esketamine (35, 36). In this regard, resistance by clinicians may be encountered to the detriment of proved clinical effectiveness in TRD. Based on such premises, we carried out a narrative review of the available literature on the most common “misconceptions” and “stereotypes” associated with esketamine use; for each false myth, we provide a list of “good reasons” for disconfirming such stereotypes.

## Methods

The keywords “esketamine”, “treatment resistant depression”, “depression”, “myth”, “mythology”, “pharmacological treatment”, and “misunderstanding” were entered in PubMed, ISI Web of Knowledge, Scopus and Medline. Terms and databases were combined using the Boolean search technique, which consists of a logical information retrieval system (two or more terms combined to make searches more restrictive or detailed). The search strategy has been limited from March 2019, when the US Food and Drug Administration (FDA) approved the use of esketamine for the treatment of treatment-resistance depression (TRD), to March 2024. The following criteria were considered for including papers in the present narrative review: 1) papers written in English; 2) papers focused on the use of esketamine as add-on treatment for TRD patients; 3) focus on prevalence of TRD and/or on side effects of esketamine treatment and/or risk of addiction due to esketamine use and/or rules of clinical practice needed for administering esketamine.

## Results from the narrative clinical review

Based on the search strategy, selected studies were used for counteracting the common false myths reported in clinical practice about the use of esketamine for the treatment of patients with TRD.

The most common false myths are the following: 1) the prevalence of TRD is low in clinical practice; 2) no specific clinical features characterize the individual patient with TRD; 3) TRD cannot be predicted before its clinical manifestation; 4) patient candidate to esketamine treatment must have reported nonresponse to either SSRIs or SNRIs; 5) patient candidate to esketamine treatment must be affected only from MDD; 6) patient treated with esketamine will report side effects, including dissociation and agitation; 7) esketamine is associated with high risk of addiction; 8) esketamine treatment requires long period of observation, with adequate room and many healthcare professionals involved in the administration procedure (**Table 1**).

**Table 1:** The most common false myths and facts regarding TRD and esketamine treatment.

Myths	Facts
The prevalence of TRD is low	TRD is a common clinical condition
No specific clinical features characterize the individual patient with TRD	The individual patient with TRD has specific clinical characteristics
TRD cannot be predicted before its clinical manifestation	Numerous clinical predictors allow to detect patients at high risk of TRD
Patient eligible for esketamine treatment must have reported nonresponse to either SSRIs or SNRIs	Patient eligible for esketamine treatment can be nonresponse to any class of antidepressants
Patient candidate to esketamine treatment must be affected only from depression	No psychiatric comorbidity can contraindicate esketamine treatment
Patient treated with esketamine will report side effects, including dissociation and agitation	Dissociation is not very frequent among side effects
Esketamine is associated with high risk of addiction	Potential addiction of esketamine is not commonly experienced by most part of patients
Esketamine treatment requires long period of observation, with adequate room and many healthcare professionals involved in the administration procedure	Esketamine treatment can be managed in outpatient unit, with the assistance of a few professionals

**Myth 1**: The prevalence of TRD is low

**Fact 1**: TRD is a common clinical condition

The Sequenced Treatment Alternatives to Relieve Depression (STAR*D) trial (37) found a cumulative remission rate of 67% throughout four acute treatment steps, while a TRD prevalence of up to 55% was detected in a cross-sectional study focused on primary care in United Kingdom (38). In more recent years, Liu et al. (39) found lower rates (5.8% and 6.0%) by analyzing data from two large databases encompassing almost 600,000 patients taking medications for depression in the United States, where a 12-month prevalence of 30.9% was also found in four claims studies (40). A similar French research detected 25.8 people suffering from TRD per 10,000 patients (41). Furthermore, TRD proportion was estimated to be 4.2% in Italy (42), 24.4% in Israel (43) and 19.6% in Thailand (44). Although prevalence data are heterogeneous, the common element is that TRD is quite frequent in ordinary clinical practice. Clinicians should be aware of the characteristics of TRD as well as of the different therapeutic strategies for managing patients suffering from TRD.

However, exact prevalence rate of TRD cannot be estimated due to the lack of a consensus definition and due to the different settings where patients can be treated (i.e., primary care, outpatient units, inpatient unit, academia) (14, 45, 46).

**Myth 2**: No specific clinical features characterize the individual patient with TRD

**Fact 2**: The individual patient with TRD has specific clinical characteristics

TRD is a clinical condition associated with high levels of social and personal burden (47), requiring half of expenditure for medical treatment of major depression in the United States (about $92.7 billion per year) (40). Patients with TRD experience significant impairment in psychosocial functioning, poor levels of quality of life, and adverse health outcomes (48–52). Hospitalization rate and emergency department utilization were found to be more than twice in TRD patients in comparison with general population (50, 53), with also significantly longer hospital stay (36% more) and higher costs (54). When compared with treatment-responding subjects, TRD patients reported more prevalent hypertension, hypothyroidism and chronic pulmonary disease (55), as well as substance use, anxiety, insomnia and pain (49). TRD patients have higher level of brain aging compared to responders (56). Furthermore, higher mortality risk (7-16 deaths per 1000 patients in 5 years) and mortality rates have been found (57–60). Compared with treatment-responsive patients, individuals with TRD are twice as likely to attempt suicide, showing a rate of 30% (61, 62).

**Myth 3**: TRD cannot be predicted before its clinical manifestations

**Fact 3**: Numerous clinical predictors allow to detect patients at high risk of TRD

Several variables have been studied as potential predictors of TRD. A European multicentric study performed on 702 patients with depression (63) detected significant association between TRD and comorbid panic disorder (OR: 3.2), anxiety (OR: 2.6), suicidal risk (OR: 2.2), social phobia (OR: 2.1), young age of onset (OR: 2.0), personality disorder (OR: 1.7), symptom severity (OR: 1.7), history of multiple hospitalizations (OR: 1.6), nonresponse to the first antidepressant taken (OR: 1.6), melancholia (OR: 1.5), and recurrent episodes (OR: 1.5). Severity and length of depressive episode, risk of suicide, psychotic symptoms, comorbid anxiety, non-response to previous antidepressants, recurrence and hospitalization were confirmed in association with TRD (64, 65), alongside with antidepressants at higher doses (66). Moreover, among physical health problems cardiovascular disease, pain and thyroid problems were most commonly reported to be associated, as well as female gender among sociodemographic variables (67). Few studies also tested the association between TRD and specific candidate genetic factors, but no specific biomarkers have been identified so far (68).

**Myth 4**: Patient eligible to esketamine treatment must have reported nonresponse to either SSRIs or SNRIs

**Fact 4**: Patient eligible for esketamine treatment can be nonresponse to any class of antidepressants

Both FDA (24) and EMA (23) approved esketamine treatment for patients with depression who had tried at least two different antidepressants without gaining benefits. In this regard, there is no specific mention of SSRIs and/or SNRIs in both approval release documents, so that failure of antidepressant treatment should be intended in general, also involving other classes (e.g., tricyclics, monoamine oxidases inhibitors, or dopamine/norepinephrine modulators, atypical antidepressants). Instead, it is worth mentioning that a SSRI or SNRI is specifically required to be used in combination with esketamine treatment. In a comparative study conducted in Italy (69), more than half of unipolar and bipolar TRD patients were taking other antidepressants besides SSRIs or SNRIs before starting esketamine. As well, no specification of class was provided regarding antidepressants taken by TRD subjects enrolled by Estrade et al. (70).

**Myth 5**: Patient candidate to esketamine treatment must be affected only from depression

**Fact 5**: No psychiatric comorbidity is a contraindication to esketamine treatment

TRD is a clinical condition often occurring with other comorbid psychiatric disorders, such as anxiety, obsessive compulsive disorder, attention-deficit/hyperactivity disorder, substance use disorder as well as self-harm behavior, fatigue, chronic pain, and insomnia (58, 71–74). In the real world, clinicians deal with patients suffering from TRD with other symptoms in comorbidity, which might benefit from esketamine treatment. No contraindications have been pointed out in release documents issued by FDA and EMA (23, 24). Furthermore, esketamine’s effectiveness was investigated in TRD patients with comorbid anxiety (75), post-traumatic stress disorder (76), and substance use disorder (77). The use of esketamine for treating patients with TRD and comorbid obsessive-compulsive disorder (78) and anorexia nervosa (79) has been described as well. Esketamine combined with an oral antidepressant has been approved in the United States for managing depression with acute suicidal ideation or behavior (80, 81), and in Europe for dealing with psychiatric emergencies in adults affected from depression.

**Myth 6**: Patient treated with esketamine will definitely experience dissociation and agitation

**Fact 6**: Dissociation is not very frequent among side effects

Dissociation is a complex construct defined as a “disruption and/or discontinuity in the normal integration of consciousness, memory, identity, emotion, perception, body representation, motor control, and behavior” (1). It encompasses depersonalization, derealization, illusions and distortion of time, which may be experienced within a few hours and mostly at a non-severe degree by 11.1-31.4% of people treated with esketamine (28). The meta-analysis by Yang et al. (82) found an overall relative risk of developing dissociation of 4.54 (*p*<.00001) among patients using esketamine when compared with placebo group. This value resulted almost twice (RR: 8.06, *p*<.00001) in the subgroup taking the dosage of 56 mg. The SUSTAIN-2 trial (83) reported dissociation rate of 23.4% during the 4-week induction period and of 18.7% during the 48-week maintenance phase. A *post-hoc* analysis found a prevalence of dissociation of 14.3% in patients forty minutes later the administration of the first dose of esketamine (84). The findings from the SUSTAIN-3 trial (85) showed dissociation in 24.4% of participants, 99.8% of whom resolved this condition during the same day of drug administration. In the real world, dissociative symptoms were detected in 39.7% of subjects (86). Causal role of dissociation in improving depressive symptoms was not consistently found (87–89). Trait dissociation, assessed through the *Dissociative Experience Scale (DES)* (90), was proved to be a significant predictor for the development of dissociation as side effect. Therefore, the DES should be used as potential screening tool for identifying patients at higher risk for developing dissociation.

Psychomotor agitation is not commonly reported as a side effect of esketamine treatment. In the REAL-ESK study (86), only one case of severe agitation was recorded among 116 treated subjects. Furthermore, a case report referring to a patient experiencing agitation and dissociation due to esketamine was described by Pereira and colleagues (91), who managed this condition throughout non-pharmacological approach.

**Myth 7**: Esketamine is associated with high risk of addiction

**Fact 7**: Potential addiction of esketamine is not commonly experienced by majority of patients

Potential addiction induced by intranasal esketamine is similar to that derived from intravenous racemic ketamine in non-dependent drug users (92). Although this aspect represents a concern for clinicians, lack of validated quantitative assessment of potential addiction in TRD patients treated with esketamine has contributed to limit evidence. Wang et al. (93) developed a visual analog scale for assessing esketamine craving and drug likeability, intended as a predictor of potential addition (94). The risk of esketamine addiction does not affect all patients equally (95). Moreover, slow de-tritation of esketamine and combined use of bupropion were suggested for managing drug-seeking and craving behaviors (34).

**Myth 8**: Esketamine treatment requires long period of observation, with adequate room and many healthcare professionals involved in the administration procedure

**Fact 8**: Esketamine treatment can be managed in outpatient unit, with the assistance of a few professionals

Esketamine treatment requires some specific conditions to be met to ensure patients monitoring and comfort. Administration should be performed in a peaceful room of hospital or outpatient unit, in which bed or chair allows patients to rest. The possibility to adjust the lighting also would be an optimal option. Sphygmomanometer and handkerchiefs are essential tool to have available. Patients have to come in the morning on an empty stomach. Esketamine is auto-administered through a nasal spray device containing 28 mg per 200 μl of vehicle solution (2 sprays). Before administration, patients are asked to clean their nose and recline their head to 45°. Blood pressure monitoring is required before and forty minutes after the last administration (20). People suffering from high blood pressure (more than 140/90 mmHg in adults; more than 150/90 mmHg in the elderly) have to be treated previously, as esketamine treatment can only start when blood pressure levels are within normal range. After monitoring by 60-90 minutes, in the absence of any problems patients can be discharged. Although they can also go home alone, they are advised not to drive the car until the next day.

## Discussion

The present narrative review aims at counteracting false myths regarding TRD and esketamine treatment by providing the most recent and updated evidence available.

TRD represents a complex clinical condition as confirmed by the lack of a consensus definition and clear epidemiological data (14, 96–99). According to EMA conceptualization (23), depression can be defined “resistant to treatment” if at least two antidepressants failed to improve depressive symptoms, despite their use at right dose, for adequate period and with adequate patient’s compliance. Therefore, many clinical conditions labelled as “depressions difficult to treat” do not fully satisfy criteria for TRD and they may not benefit from treatments approved for TRD. Some clinical features might be useful in detecting real condition of TRD, and the identification of clear predictors of TRD can be helpful for optimizing diagnosis and subsequently therapy. It has to be noted that esketamine is approved for treatment-resistant depression (TRD) and emergency suicidality only. However, recent trials have confirmed its efficacy also in patients suffering from bipolar disorder, with an actual depressive phase (69), but this use remains off-label and clinicians should carefully evaluate the risk/benefit ratio in administering such medication to patients with different clinical conditions. Although these positive results are encouraging, further longitudinal studies, designed with a rigorous methodology, are required.

Esketamine represents an additional tool in the clinicians’ therapeutic armamentarium for treating MDD and TRD. Clinical efficacy has been proved both in experimental and real-world settings. Superiority of esketamine combined with oral antidepressant compared to placebo plus oral antidepressant was found in the short-term by Popova et al. (100), unlike Fedgchin et al. (101) and Ochs-Ross et al. (102). In the long-term treatment, esketamine is effective in terms of significant reduction of depressive symptoms (83). Moreover, in the long-term maintenance study, adult patients with TRD treated with a continue use of esketamine report a significant delay in time to relapse compared with placebo, both considering stable remitters and stable responders (103). It is relevant to consider that no potential risk for abuse has been detected in the long-term treatment (i.e., up to one year from treatment) (104).

In the real world, significant improvements in terms of depressive symptoms and remission rates were reported after three months from the start of treatment (86), also in subjects affected by bipolar TRD (69), and in elder patients who however showed high levels of side effects (105).

Esketamine represents an important novelty among pharmacological treatments for patients with MDD, having an innovative mechanism of action (106). Indeed, depression has traditionally been conceptualized as a disorder underlying by an alteration in the neurotransmission pathways of serotonin, norepinephrine and dopamine pathways. Esketamine works as non-selective, non-competitive antagonist of NDMA receptor, determining subsequent activation of AMPA and intracellular cascades (31, 32). Higher levels of BDNF and synaptic plasticity represent positive effects. Therefore, esketamine has a specific target on a new pathway, which is represented by the glutamatergic system. However, given its similar pharmacological profile and the extensive literature on its safety and tolerability, it is crucial to briefly mention ketamine (107). Many randomized controlled trials have confirmed the acute efficacy of ketamine in patients with TRD, although only a few data come from the real-world practice. A recent systematic review (26) found that ketamine has a substantial antidepressant effect, although its effectiveness varies significantly across patients. Moreover, a recent study by Gałuszko-Węgielnik et al. (108) found that ketamine is an effective add-on treatment to standard of care for people with treatment-resistant depression presenting psychotic features. Ketamine is administered as intravenous infusion and the subsequent monitoring revealed no exacerbation of psychotic symptoms in short and long-term observation, while stable remission and fast antisuicidal effect was found. However, these data should be carefully considered since the rates of recreational use of ketamine is increasing and the potential addiction to ketamine shares the same neurobiological pathway of its clinical effectiveness in treating patients suffering from TRD (109).

Taking esketamine requires a safe setting, where healthcare professionals can monitor patient’s response in terms of side effects for up to 90 minutes. Dizziness, nausea, dissociation, headache, dysgeusia, vertigo, somnolence, hypoesthesia and vomiting were reported as common side effects (110). Usually, they appear at mild or moderate degree of severity, are dose-dependent, and last only in the same day of esketamine administration. When they are severe, adjunctive treatments, and/or treatment pause or interruption should be considered (111–113). Discontinuation rate due to adverse effects in clinical trials has been estimated in about 5% of cases (85). The most relevant limitation is using esketamine is related to patients at high risk of aneurysm, and those with history of cerebral bleeding or heart attack (20). Assisted administration and monitored setting may also be helpful to promptly detect any potential risk of addiction.

Basing on patients’ age, recommended dosage consists of one or two puffs in each nostril at day 1, while up to three sprays per nostril can be administered twice a week during the following 4 weeks. Depending on patient’s conditions, treatment can be performed once a week for 4 weeks and once or two times per week up to 6 months. This strict schedule may appear a limitation for patients, but real-world study does not mention this aspect among the reasons of esketamine discontinuation (69).

Intranasal administration is unusual in psychiatric setting. Indeed, consolidated use of tablets, capsules and drops formulations has allowed the patient to take antidepressant therapy in comfort and autonomy. Furthermore, repeated and intermittent nasal sprays encouraged researchers to investigate olfactory functionality and nasal mucosa of patients, who seem to well tolerate this practice also in the long term (114, 115).

Dissociative effects and the potential addictive effects of esketamine treatment are among the main concerns related to the use of esketamine in clinical practice.

As regards dissociative effects, these are experienced as feelings of disconnection from the reality, and are reporting in up to 40% of subjects taking esketamine in the real world setting, resolving within the same day of administration. Although a causal role of dissociation in improving depressive symptoms could be hypothesized, Ballard and Zarate (87) showed that it is not necessary to determine antidepressant effects of ketamine and derived medication. Moreover, the potential addiction from this drug resulted to involve patients treated with esketamine (95).

The present study has some limitations, which must be acknowledged. First, this is not a systematic review, but rather a narrative review which is more in line with the scope of the paper. It may be that relevant studies on esketamine have been omitted, but this was due to the need to identify papers related to the false myths addressed here. In fact, narrative reviews are a specific type of review in which researchers can pursue an extensive description and interpretation of previously published papers on a chosen topic. The description of the search strategy and selection criteria should be considered a major strength of the present paper. We believe that this approach has been appropriate for the topic of “myth and facts” related to the use of esketamine in ordinary routine clinical practice.

Another limitation is the inclusion of papers published in English only, which may have led to the exclusion of some papers/clinical experiences carried out in different countries with different languages.

## Conclusions

TRD represents a challenging clinical condition, which needs to be adequately identified and diagnosed in order to achieve patient’s full recovery. Esketamine has been proved as an effective medication to treat TRD, although it requires precautions. Evidence can inform clinical practice, in order to offer this innovative treatment to all patients with TRD.

Although esketamine is an innovative treatment for the management of TRD patients, available data clearly confirm the efficacy, safety and good tolerability profile of this medication.

## Author contributions

MDV: Investigation, Writing – original draft. VM: Conceptualization, Writing – review & editing. BDR: Methodology, Writing – review & editing. EA: Methodology, Writing – review & editing. AD’A: Methodology, Writing – review & editing. AV: Investigation, Writing – original draft. ML: Methodology, Writing – review & editing. GS: Conceptualization, Writing – original draft. AF: Supervision, Writing – original draft, Writing – review & editing.
